# Risk Factors for COVID-19 and Respiratory Tract Infections during the Coronavirus Pandemic

**DOI:** 10.3390/vaccines12030329

**Published:** 2024-03-19

**Authors:** Laurynas Mockeliunas, Rob C. van Wijk, Caryn M. Upton, Jonathan Peter, Andreas H. Diacon, Ulrika S. H. Simonsson

**Affiliations:** 1 Department of Pharmaceutical Biosciences, Uppsala University, 751 24 Uppsala, Sweden; 2 TASK, Cape Town 7500, South Africa; 3 Allergy and Immunology Unit, University of Cape Town Lung Institute and Division of Allergy and Clinical Immunology, University of Cape Town, Cape Town 7700, South Africa

**Keywords:** risk factors, respiratory tract infections, COVID-19, time-to-event analysis, pharmacometrics

## Abstract

(1) Background: Some individuals are more susceptible to developing respiratory tract infections (RTIs) or coronavirus disease (COVID-19) than others. The aim of this work was to identify risk factors for symptomatic RTIs including COVID-19 and symptomatic COVID-19 during the coronavirus pandemic by using infection incidence, participant baseline, and regional COVID-19 burden data. (2) Methods: Data from a prospective study of 1000 frontline healthcare workers randomized to Bacillus Calmette–Guérin vaccination or placebo, and followed for one year, was analyzed. Parametric time-to-event analysis was performed to identify the risk factors associated with (a) non-specific symptomatic respiratory tract infections including COVID-19 (RTIs+COVID-19) and (b) symptomatic RTIs confirmed as COVID-19 using a polymerase chain reaction or antigen test (COVID-19). (3) Results: Job description of doctor or nurse (median hazard ratio [HR] 1.541 and 95% confidence interval [CI] 1.299–1.822), the reported COVID-19 burden (median HR 1.361 and 95% CI 1.260–1.469 for 1.4 COVID-19 cases per 10,000 capita), or a BMI > 30 kg/m^2^ (median HR 1.238 and 95% CI 1.132–1.336 for BMI of 35.4 kg/m^2^) increased the probability of RTIs+COVID-19, while positive SARS-CoV-2 serology at enrollment (median HR 0.583 and 95% CI 0.449–0.764) had the opposite effect. The reported COVID-19 burden (median HR 2.372 and 95% CI 2.116–2.662 for 1.4 COVID-19 cases per 10,000 capita) and a job description of doctor or nurse (median HR 1.679 and 95% CI 1.253–2.256) increased the probability of developing COVID-19, while smoking (median HR 0.428 and 95% CI 0.284–0.648) and positive SARS-CoV-2 serology at enrollment (median HR 0.076 and 95% CI 0.026–0.212) decreased it. (4) Conclusions: Nurses and doctors with obesity had the highest probability of developing RTIs including COVID-19. Non-smoking nurses and doctors had the highest probability of developing COVID-19 specifically. The reported COVID-19 burden increased the event probability, while positive SARS-CoV-2 IgG serology at enrollment decreased the probability of RTIs including COVID-19, and COVID-19 specifically.

## 1. Introduction

Coronavirus disease (COVID-19) is an infectious disease caused by severe acute respiratory syndrome coronavirus 2 (SARS-CoV-2). The COVID-19 pandemic dramatically affected the globe with more than 770 million confirmed COVID-19 cases and 6.9 million deaths reported globally from the time of virus emergence in December 2019 to January 2024 [1]. Coronavirus disease is a respiratory tract infection (RTI), which is defined as any infectious disease of the upper or lower respiratory tract.

Despite the rapid transmission of the virus, some individuals have a higher probability of contracting COVID-19 than others. Large heterogeneity is typically present among individuals, especially when it comes to the development of many widespread viral and bacterial infections, like influenza [2] or tuberculosis [3]. The same is the case for COVID-19; thus, the importance of identifying vulnerable groups, more susceptible to infection with SARS-CoV-2 and other infectious agents causing RTIs, is of importance. Some of the risk factors for the probability of contracting COVID-19 identified in previous studies include sex [4,5,6,7,8], age [6,7,8,9], higher body mass index (BMI) [4,6,8], higher glycated hemoglobin levels associated with diabetes [4,8], smoking [4,8], and use of blood pressure medications associated with hypertension [4,5,8,10]. Information about risk factors for the flu and other common respiratory diseases is available from government agencies like the Centers for Disease Control and Prevention (CDC) [2] and scientific research, where age [11,12,13,14], chronic medical conditions (chronic obstructive pulmonary disease (COPD) [15], diabetes mellitus [13], cardiovascular disease [13], asthma [16,17], obesity [11,12,18,19,20,21]), sex [11,13,15,16], smoking [12,18,22,23], being a part of the nursing personnel, a nurse, or a doctor [24,25], and others are identified as risk factors for RTIs, but it is unknown if the risk factors for RTIs before the COVID-19 pandemic were the same as during the coronavirus pandemic.

Enforced protective measures, like hand hygiene, masks, and social distancing globally resulted in a decrease in both COVID-19 and RTI cases [26]. Despite this, both COVID-19 and other RTIs co-existed during the pandemic, further complicating medical care.

Before COVID-19 vaccines became available, repurposing already existing vaccines such as Bacillus Calmette–Guérin (BCG) and measles vaccines was proposed as a stop-gap measure based on the hypothesis of a non-specific trained immunity against a range of pathogens that might include coronaviruses. For this reason, a trial in South Africa was conducted to evaluate the impact of BCG (re)vaccination on morbidity and mortality due to COVID-19 in healthcare workers (ClinicalTrials.gov Identifier: NCT04379336, accessed on 18 January 2024) [27]. Considering the detailed data collected in the trial of the incidence of both non-COVID-19 RTIs and COVID-19, it became possible to identify the risk factors for RTIs including COVID-19 (RTIs+COVID-19), and COVID-19 specifically. This knowledge may be relevant for prioritizing protective measures in high-risk groups in future pandemics.

The aim of this work was to identify risk factors for symptomatic RTIs including COVID-19 and symptomatic COVID-19 during the coronavirus pandemic by using infection incidence, participant baseline, and regional COVID-19 burden data.

## 2. Materials and Methods

### 2.1. Study Design and Data Sources

A randomized, double-blind, placebo-controlled trial with adult healthcare workers, who were expected to be highly exposed to COVID-19 [27], was described previously. Briefly, 1000 participants were enrolled in the trial, with a ratio of 1:1 for BCG and placebo. The trial started recruiting early in the pandemic, and the first individual was enrolled 4 May 2020. Enrollment ended 23 October 2020, and the last follow-up occurred 18 October 2021.

Participant characteristics of interest are presented in Table 1. Gender (biological), age, BMI, job description (doctor, nurse, or essential workers defined as other personnel including security and support staff), smoking status, South African district information, and medical history of comorbidities including asthma, hypertension, and cardiovascular disease were collected at enrollment through the self-reporting of the participants. Testing for SARS-CoV-2 antibodies was performed at week 0 (enrollment), week 10, week 26, and week 52. Tuberculosis (TB) infection status was determined using interferon-gamma release assay (IGRA, a proxy for the presence of latent TB infection) at enrollment and week 52. Obtaining a SARS-CoV-2-specific vaccine became possible for the study population in February 2021. Information about RTI (all RTIs including COVID-19) occurrence was collected during regularly performed follow-ups.

The first event outcome analyzed was non-specific symptomatic RTIs including COVID-19 (onwards referred to as RTIs+COVID-19). The second event outcome analyzed was symptomatic RTIs confirmed as COVID-19 using a PCR or antigen test (onwards referred to as COVID-19).

PCR and antigen tests for COVID-19 were initiated by the participants themselves in the presence of symptoms throughout the trial, while testing for SARS-CoV-2 antibodies was performed according to the protocol. SARS-CoV-2 antibodies could be positive without a history of symptoms. Asymptomatic COVID-19 was not included as an event in the analysis. In each participant, only the first RTIs+COVID-19 or COVID-19 event was analyzed, while a participant might have experienced more than one event.

**Figure 1:**
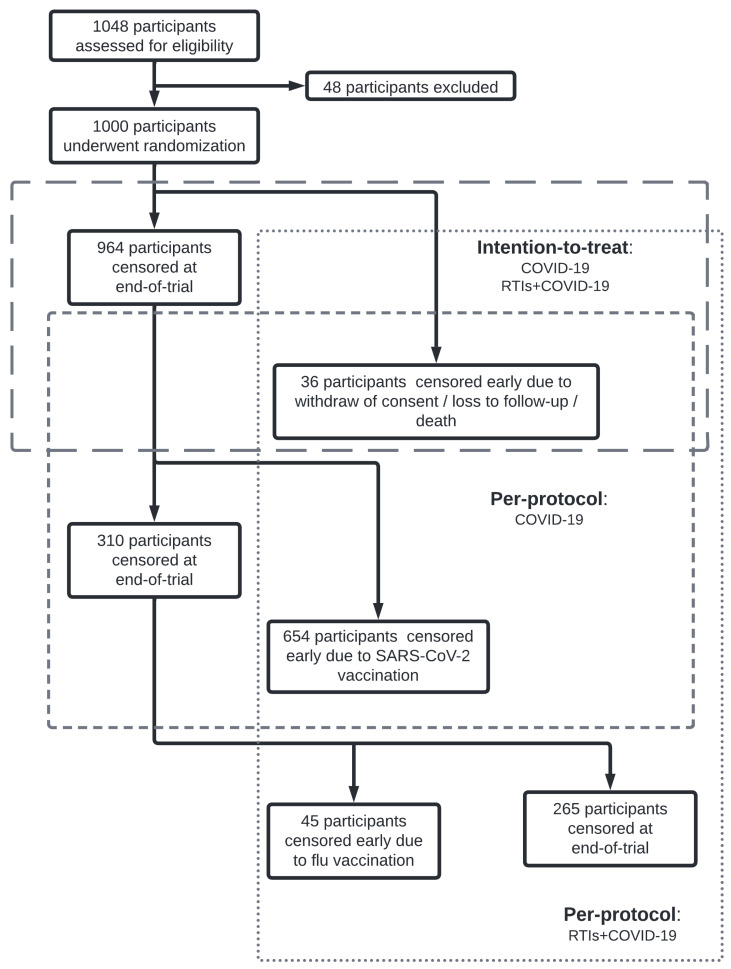
Schematic representation of the number of participants enrolled and censored due to different reasons throughout the trial. Both intention-to-treat (ITT) and per-protocol (PP) datasets are shown for both COVID-19 diagnosed with a positive PCR or antigen test (COVID-19) and non-specific respiratory tract infections including COVID-19 (RTIs+COVID-19) analyses. Participants were censored early due to withdrawal of consent/loss to follow-up/death (applicable for both ITT and PP datasets), administration of SARS-CoV-2 vaccine (PP dataset; COVID-19 and RTIs+COVID-19 endpoints) and flu vaccine (PP dataset; RTIs+COVID-19 endpoint), or when reaching the end of the trial.

In the intention-to-treat (ITT) analysis, participants were censored due to withdrawal from the trial, loss to follow-up, death, or due to the final visit of the trial (Figure 1). In the per-protocol (PP) analysis, in addition to the ITT censoring, the participants in the COVID-19 group were censored early due to SARS-CoV-2-specific vaccination, and those in the RTIs+COVID-19 group due to SARS-CoV-2 and/or flu vaccinations during trial follow-up.

The reported COVID-19 burden for each district of interest, the City of Cape Town Metro and the Garden Route, corresponding to the Cape Town (TASK Central and University of Cape Town, South Africa) and TASK Eden sites, respectively, was extracted from the Western Cape Government COVID-19 dashboard [28]. Total cumulative COVID-19 cases (daily resolution) were transformed into new daily COVID-19 cases and rescaled per 10,000 capita for each respective district of interest, termed COVID-19 burden, and matched using the record date [29].

### 2.2. Statistical Analysis

Parametric time-to-event (TTE) analysis was performed to analyze data. Exponential (Equation (1)), Weibull (Equation (2)), Gompterz (Equation (3)), and log-normal (Equation (4)) hazard distributions were evaluated as baseline hazard functions $h_{0}\left(t\right)$ [30]. Initial estimates for the models were selected using the Shiny Application for parametric time-to-event analysis [30].

(1) $$h_{0}=\lambda$$

(2) $$h_{0}=\lambda \alpha {\left(\lambda t\right)}^{\alpha -1}$$

(3) $$h_{0}=\lambda e^{\alpha t}$$

(4) $$h_{0}=\frac{{\left(\sigma t\sqrt{2\pi }\right)}^{-1}e^{\left(-\frac{1}{2}{\left(\frac{\ln t-\mu }{\sigma }\right)}^{2}\right)}}{1-\Phi \left(\frac{\ln t-\mu }{\sigma }\right)}$$

where *t* is time in days, *λ* is a scale parameter, *α* is a shape parameter, *μ* is the mean, *σ* is the standard deviation of a log-normal distribution, and Φ is the standard normal cumulative distribution function.

After the baseline hazard distribution was defined, stepwise covariate modeling with adaptive scope reduction [31] was performed to identify statistically significant risk factors. Gender (biological), age, BMI, job description, smoking status, self-reported expected exposure to COVID-19 patients, SARS-CoV-2 IgG serology at enrollment, reported COVID-19 burden, medical history of comorbidities like diabetes mellitus, hypertension, asthma, and cardiovascular disease, occurrence of a positive IGRA result at enrollment and week 52, and the IGRA conversion from negative at baseline to positive and from positive at baseline to negative were evaluated in stepwise covariate modeling. The same risk factors were investigated in both analyses.

The likelihood ratio test was used to compare nested models in stepwise covariate modeling, assuming a χ^2^-distribution. In the forward inclusion step, the *p*-value was set to 0.05, and in the backward deletion step, a *p*-value of 0.01 was used. Risk factors were incorporated into the hazard function as follows (Equation (5)):

(5) $$h\left(t\right)=h_{0}\left(t\right)\times e^{\beta_{1}X_{1}+\cdots +\beta_{n}X_{n}}$$

where $h_{0}\left(t\right)$ is the baseline hazard function, and $\;\beta_{n}\;$ is the coefficient describing the effect of a covariate (defined as $\;X_{n}\;$ in the equation).

Hazard ratios (HRs) were derived to compare the log-linear effect of statistically significant risk factors. Sampling importance resampling [32] with 2000 samples was performed to derive 95% confidence intervals (CI) for forest and cumulative probability plots.

The cumulative probability within one year for each outcome was calculated using the sampling importance resampling results. Every combination of statistically significant risk factors was derived. For categorical risk factors, the influential feature was either present or absent. Continuous risk factors were categorized, and several different values from the range were selected.

Throughout model development, model fit was evaluated using the objective function value (OFV), kernel-based visual hazard comparison (kbVHC) [33], and the Kaplan–Meier visual predictive check (VPC).

### 2.3. Software

Data preparation and graphical analysis following a reproducible workflow [34] were performed using R (v. 4.2.2, R Foundation for Statistical Computing, Vienna, Austria) [35] and the RStudio user interface (v.2023.6.0.421) [36]. Non-linear mixed-effects modeling with a first-order method with a likelihood (NONMEM v. 7.5.0) [37] was used through Perl-speaks-NONMEM (PsN) (v. 5.3.0) (https://uupharmacometrics.github.io/PsN/, accessed on 18 January 2024). The integration of the cumulative probability of RTIs+COVID-19 or COVID-19 was performed using the *flexsurv* R-package (version 2.2.1) [38]. The Shiny Application was utilized to assess hazard functions in the parametric time-to-event analysis and to support model development [30].

## 3. Results

### 3.1. Respiratory Tract Infections including COVID-19

**Figure 2:**
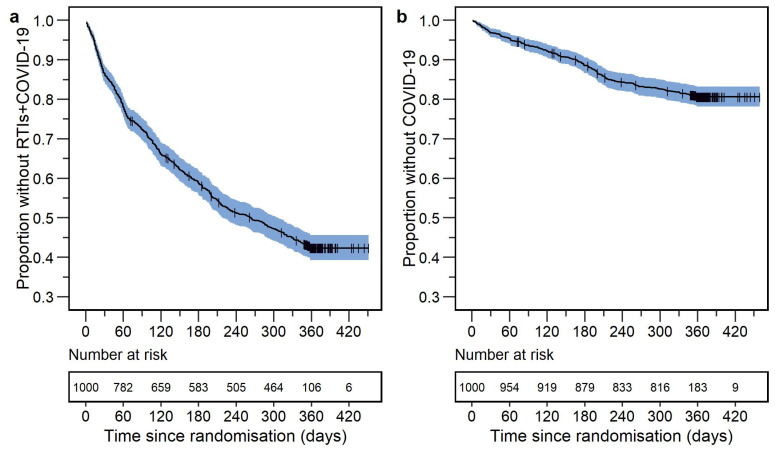
Kaplan–Meier plots of the data (enhanced *y*-axis) for the time-to-first event in the intention-to-treat analysis for (**a**) respiratory tract infections including COVID-19 (RTIs+COVID-19) and (**b**) COVID-19. The shaded area around the Kaplan–Meier curve represents the standard error, obtained by the Greenwood method [39]. Vertical dashes represent censoring, while a step down represents an event. The number at risk below the figure presents the number of participants still in the trial.

**Figure 3:**
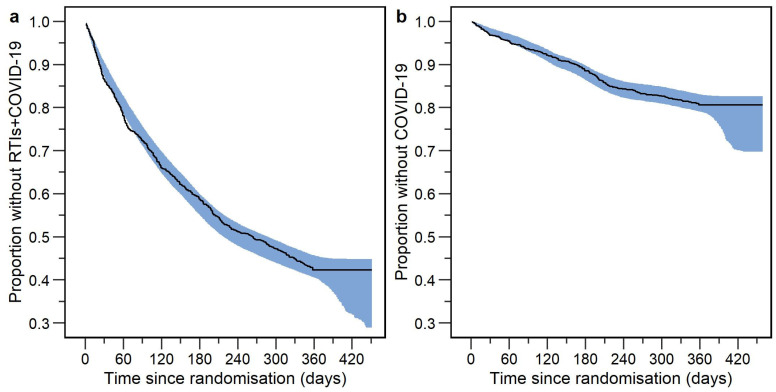
Visual predictive checks for the final models (enhanced *y*-axis) for the time-to-first event in the intention-to-treat analysis for (**a**) respiratory tract infections including COVID-19 (RTIs+COVID-19) and (**b**) COVID-19. The black line in the VPC represents observed data, while the shaded area represents the 95% confidence interval based on 1000 simulations using the final parametric time-to-event model.

In the ITT analysis, 569 participants out of 1000 (56.9%) had an event. The Kaplan–Meier plot of the data is shown in Figure 2, panel a. The baseline hazard function was best described by the Gompertz function, and the hazard decreased over time. The final model contained four statistically significant risk factors. The final parameter estimates are presented in Table 2. A visual predictive check for the final model is shown in Figure 3, panel a. The final model code is presented in Supplementary Materials Text S1.

**Figure 4:**
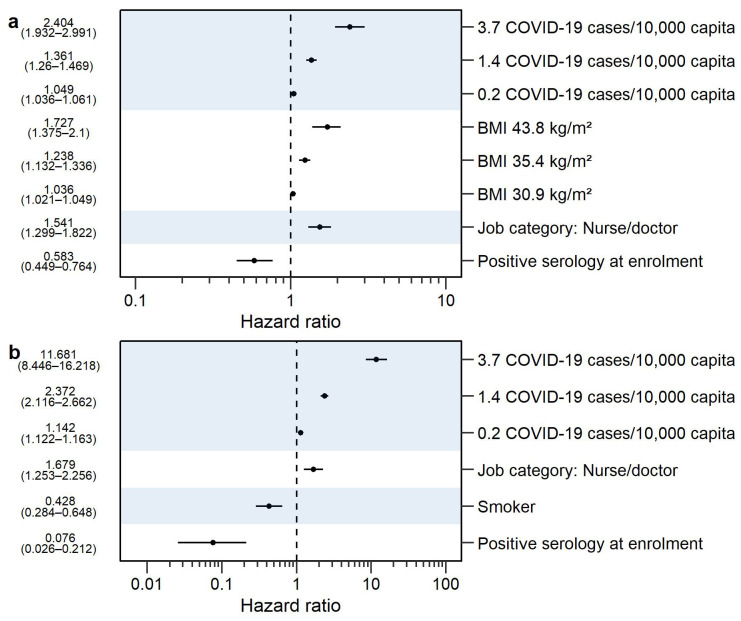
Forest plots for the statistically significant risk factor effects on cumulative probability in the intention-to-treat analysis for (**a**) respiratory tract infections including COVID-19 (RTIs+COVID-19) and (**b**) COVID-19. Risk factor effects are expressed as hazard ratios (HRs), the circle represents the median HR, while the whiskers represent a 95% confidence interval. The covariate values on the *y*-axis were derived from the observed data as either the unique categories of the categorical covariates or as the 10th, 50th, and 90th percentiles of the continuous covariates [40]. The numbers next to the forest plot represent the median HR and 95% confidence interval. Body mass index (BMI) < 30 kg/m^2^ did not affect the hazard ratio while BMI > 30 kg/m^2^ resulted in an increasing hazard ratio with an increasing covariate value.

**Figure 5:**
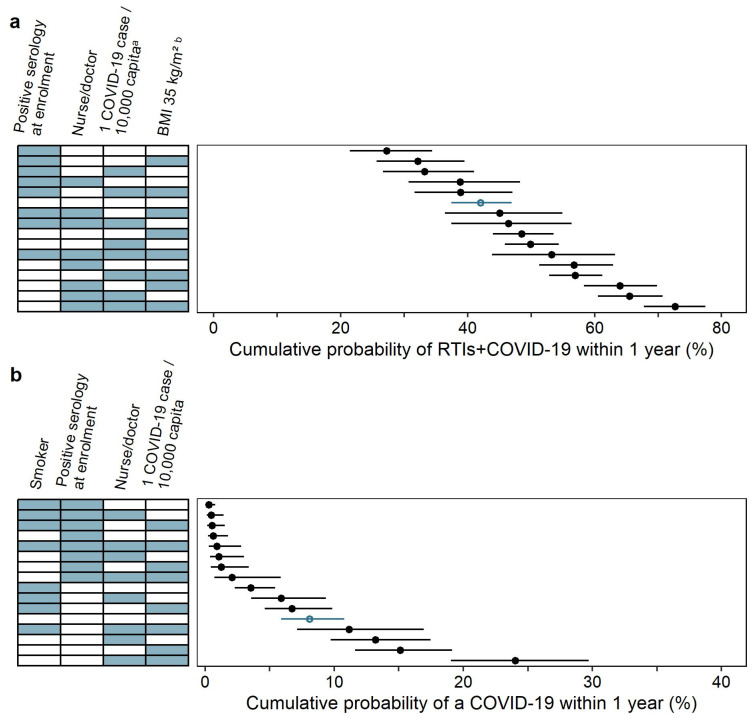
The cumulative probability of having an event within one year for different combinations of statistically significant risk factors in the intention-to-treat analysis for (**a**) respiratory tract infections including COVID-19 (RTIs+COVID-19) and (**b**) COVID-19. Blue cells represent the inclusion of a significant risk factor (named above), while white cells represent the inclusion of a reference feature (absence of the named risk factor). The circle represents the median, while the whiskers represent a 95% confidence interval. The reference cumulative probability (absence of any named risk factors) is shown in a blue empty circle. One COVID-19 case per 10,000 capita—reported COVID-19 burden, where the risk factor included was set to one case per 10,000 capita (reference: zero reported COVID-19 cases per 10,000 capita). Positive serology—corresponds to positive SARS-CoV-2 IgG serology at enrollment (reference: negative SARS-CoV-2 IgG serology at enrollment). Nurse/doctor—job category classified as a nurse/doctor (reference: essential worker). BMI 35 kg/m^2^—body mass index (BMI) corresponding to being obese (reference: BMI < 30 kg/m^2^). Smoker—indicates that the participant was a smoker (reference: non-smoker). ^a^ The continuous time-varying risk factor of the reported COVID-19 burden was treated as a categorical time-constant risk factor for the computation of cumulative probabilities, and two categories were selected: zero COVID-19 cases per 10,000 capita and one COVID-19 case per 10,000 capita. ^b^ The continuous covariate BMI was categorized into two categories for the purpose of computation of cumulative probability: BMI < 30 kg/m^2^ (indicating no impact of the risk factor on the cumulative probability) and BMI of 35 kg/m^2^ (visualizing the impact of the risk factor on the cumulative probability).

The forest plot of statistically significant risk factors is presented in Figure 4, panel a. Participants with a positive SARS-CoV-2 IgG serology status at enrollment had a lower probability of RTIs+COVID-19, compared to those with a negative serology status (median HR of 0.583), while those with a job description of nurse or doctor had a higher probability of RTIs+COVID-19 compared to essential workers (median HR of 1.541). Additionally, having BMI > 30 kg/m^2^ increased the probability of RTIs+COVID-19, as visualized for BMIs of 30.9, 35.4, and 43.8 kg/m^2^ resulting in median HRs of 1.036, 1.238, and 1.727, respectively. The parameter–covariate relationship for BMI was centered around 30 kg/m^2^, as this value is used as a cut-off value to differentiate between overweight and obese participants, which also approximated the median BMI of the trial (28.6 kg/m^2^). As indicated by hockey-stick continuous covariate parameterization, BMI < 30 kg/m^2^ had minimal influence on the probability, while the probability of RTIs+COVID-19 increased with each kg/m^2^ unit for BMI > 30 kg/m^2^, which was shown in Figure 5, panel a, for 35 kg/m^2^ (i.e., a 5-unit increase). Lastly, the reported COVID-19 burden was shown to have an influence on the probability of having RTIs+COVID-19. Here, 0.2, 1.4, and 3.7 COVID-19 cases per 10,000 capita resulted in median HRs of 1.049, 1.361, and 2.404, respectively.

The cumulative probabilities within one year for different combinations of risk factors are shown in Figure 5, panel a. Of the assessed risk factor combinations, the lowest cumulative probability of RTIs+COVID-19 was related to BMI < 30 kg/m^2^, a job category classified as an essential worker, no reported COVID-19 burden cases, and with a positive SARS-CoV-2 IgG serology status at enrollment (cumulative probability of 27.22%, 95% CI 21.48–34.43%), while the highest cumulative probability of RTIs+COVID-19 was associated with BMI of 35 kg/m^2^, a negative SARS-CoV-2 IgG serology status at enrollment, a job category classified as a nurse/doctor, and the reported COVID-19 burden of one case per 10,000 capita (cumulative probability of 72.66%, 95% CI 67.76–77.47%). The reference (defined as BMI < 30 kg/m^2^, a negative SARS-CoV-2 IgG serology status at enrollment, a job description of essential worker, and no reported COVID-19 cases) was predicted to have a cumulative probability of 42.00% (95% CI 37.44–46.97%).

**Figure 6:**
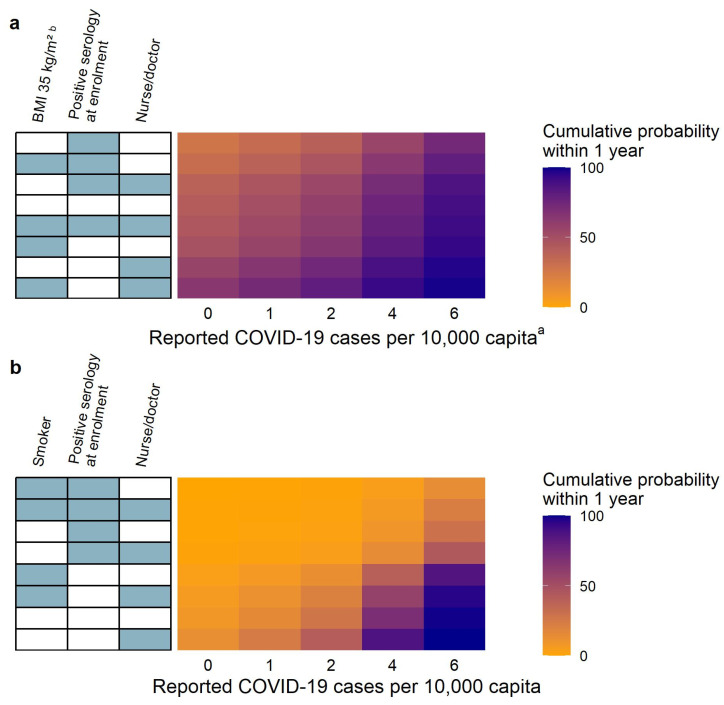
Reported COVID-19 burden influence on the cumulative probability of having an event within one year for different combinations of statistically significant risk factors in the intention-to-treat analysis for (**a**) respiratory tract infections including COVID-19 (RTIs+COVID-19) and (**b**) COVID-19. The heatmap shows the median cumulative probability for each combination of the reported COVID-19 burden and other statistically significant risk factors. The blue cells represent the inclusion of a significant risk factor (named above), while the white cells represent the inclusion of a reference feature (absence of the named risk factor). The circle represents the median, while the whiskers represent a 95% confidence interval. The reference cumulative probability (absence of any named risk factors) is shown in a blue empty circle. One COVID-19 case per 10,000 capita—reported COVID-19 burden, where the risk factor included was set to one case per 10,000 capita (reference: zero reported COVID-19 cases per 10,000 capita). Positive serology—corresponds to positive SARS-CoV-2 IgG serology at enrollment (reference: negative SARS-CoV-2 IgG serology at enrollment). Nurse/doctor—job category classified as a nurse/doctor (reference: essential worker). BMI of 35 kg/m^2^—body mass index (BMI) corresponding to being obese (reference: BMI < 30 kg/m^2^). Smoker—indicates that the participant was a smoker (reference: non-smoker).^a^ The continuous time-varying risk factor of the reported COVID-19 burden was treated as a categorical time-constant risk factor for the computation of cumulative probabilities. ^b^ The continuous covariate BMI was categorized into two categories for the purpose of computation of cumulative probability: BMI < 30 kg/m^2^ and BMI of 35 kg/m^2^.

The influence of the reported COVID-19 burden on the cumulative probability of RTIs+COVID-19 within one year for different combinations of statistically significant risk factors is shown in Figure 6, panel a. The cumulative probability of RTIs+COVID-19 increased with an increasing reported COVID-19 burden, and for the reference participant (defined as BMI < 30 kg/m^2^, with a negative SARS-CoV-2 IgG serology status at enrollment, and with a job description of essential worker), the median cumulative probability was predicted to be 42.00%, 49.89%, 58.43%, 75.58%, and 89.63% for zero, one, two, four, and six reported COVID-19 burden cases per 10,000 capita, respectively.

The results for the PP analysis, which were identical regarding both the baseline hazard function and the identified statistically significant risk factors, are presented in Supplementary Materials.

### 3.2. COVID-19

In the ITT analysis, 190 out of 1000 participants (19.0%) reported COVID-19 throughout the trial. The Kaplan–Meier plot of the data is shown in Figure 2, panel b. The Gompertz function described the baseline hazard rate the best, and the hazard rate decreased over time. The final model contained four statistically significant risk factors. The final parameter estimates are presented in Table 2. The forest plot for statistically significant risk factors is shown in Figure 3, panel b. A visual predictive check of the final model is shown in Figure 4, panel b. The final model code is presented in Supplementary Materials Text S2.

The highest HRs were associated with the reported COVID-19 burden, and HRs increased with an increasing reported COVID-19 burden, as visualized for 0.2, 1.4, and 3.7 COVID-19 cases per 10,000 capita with respective median HRs of 1.142, 2.372, and 11.681 in Figure 4, panel b. This was followed by job description, where nurses and doctors had a higher probability of contracting COVID-19 compared to essential workers (median HR of 1.679). Smokers had a lower probability of COVID-19 compared to non-smokers (median HR of 0.428), and participants with a positive SARS-CoV-2 IgG serology status at enrollment had a lower probability, compared to a negative serology status (median HR of 0.076).

The cumulative probabilities within one year for different combinations of risk factors are shown in Figure 5, panel b. Of all the assessed risk factor combinations, the lowest cumulative probability was associated with smokers, a positive SARS-CoV-2 IgG serology status at enrollment, being an essential worker, and with no reported COVID-19 cases (median cumulative probability of 0.28%, 95% CI 0.09–0.80%), while the highest cumulative probability was associated with non-smokers, a negative SARS-CoV-2 IgG serology status at enrollment, being a nurse/doctor, and with a reported COVID-19 burden of one case per 10,000 capita (median cumulative probability of 24.02%, 95% CI 19.03–29.71%). The reference (defined as a non-smoker, with a negative SARS-CoV-2 IgG serology status at enrollment, a job description of essential worker, and with no reported COVID-19 cases) was predicted to have a cumulative probability of 8.07% (95% CI 5.90–10.78%).

The influence of the reported COVID-19 burden on the cumulative probability of COVID-19 was further explored, and the impact of it is shown in Figure 6, panel b. Here, the cumulative probability increased with an increasing reported COVID-19 burden, and for the reference participant (with a negative SARS-CoV-2 IgG serology status at enrollment, a job description of essential worker, and a non-smoker) the median cumulative probability was predicted to be 8.07%, 15.11%, 27.37%, 70.09%, and 98.94% for zero, one, two, four, and six reported COVID-19 burden cases per 10,000 capita, respectively. Additionally, positive serology at baseline provided noticeable protective effects regardless of the reported COVID-19 burden as expressed in the cumulative probability.

The results for the PP analysis, which were identical regarding both the baseline hazard function and the identified statistically significant risk factors, are presented in Supplementary Materials.

## 4. Discussion

This paper presented the identified risk factors for non-specific symptomatic RTIs including COVID-19 and symptomatic RTIs confirmed as COVID-19 diagnosed with a positive PCR or antigen test, using data from a prospective study where 1000 healthcare workers were followed up for one year. The identification of the risk factors contributes to knowledge about vulnerable subpopulations at risk of developing RTIs+COVID-19 or COVID-19 at the population level. Additionally, these results shed more light on the co-existence of COVID-19 together with other RTIs.

In this work, essential workers were shown to have a lower probability of having RTIs+COVID-19 or COVID-19 compared to nurses and doctors in both analyses, likely due to nurses and doctors being in the closest proximity to patients, compared to other personnel.

Participants with positive SARS-CoV-2 IgG serology at enrollment had a lower probability of both COVID-19 and RTIs+COVID-19. As 15.3% of the participants had positive SARS-CoV-2 IgG serology at enrollment, it was possible to investigate the effect of prior SARS-CoV-2 infections. In this study, the probability of contracting SARS-CoV-2 was 91.5% lower for those who had positive SARS-CoV-2 IgG serology at enrollment, which is in accordance with results from Hall et al. [41].

The inclusion of the reported COVID-19 burden in the model provided the opportunity to describe the occurrence of events in the context of the pandemic. This allowed us to better characterize the relationship between the reported COVID-19 burden and the occurrence of events. As expected, with a higher pandemic burden, the probability of having an event increased, which is consistent with our separate analysis describing the severity of RTIs+COVID-19 over time [29].

In addition, smokers were shown to have a lower probability of contracting COVID-19 compared to non-smokers. Several studies suggest that the ‘smoker’s paradox’ is present for COVID-19 [42,43,44,45], while others have observed that smoking increases the risk of both contracting the virus and having COVID-19 [4,46]. Behavioral patterns of smokers could be influential, i.e., the disregard of low-grade symptoms like coughing, or enforced social distancing due to smoking restrictions in public spaces.

Lastly, increasing BMI above 30 kg/m^2^ was shown to be correlated with an increased probability of RTIs+COVID-19. Being overweight or obese has been shown to increase the risk of both upper and lower respiratory tract infections [20], which is in accordance with our findings.

The risk factors for RTIs+COVID-19 identified in this trial including 1000 healthcare workers are in accordance with the findings of risk factors for RTIs from the pre-COVID-19 pandemic times. In multiple studies, high BMI (>30 kg/m^2^) has been shown to be associated with various RTIs [11,12,18,19,20,21] indicating a relationship between increased BMI and a higher probability of RTIs. In addition, nursing personnel, nurses, and doctors are at a higher risk of contracting non-specific RTIs compared to other hospital personnel [24,25], which is in alignment with our findings from a COVID-19 setting. Some other pre-COVID-19 pandemic reported RTI risk factors include sex [11,13,15,16], age [11,12,13,14], smoking [12,18,22,23], and chronic medical conditions [15] (diabetes mellitus [13], cardiovascular disease [13], and asthma [16,17]), which were included in our analysis but not found to be associated with non-specific symptomatic RTIs+COVID-19. A study conducted during the pandemic identified age, sex, chronic medical conditions (diabetes mellitus, hypertension, cardiovascular disease, and chronic kidney disease), immunosuppression, and obesity as risk factors for pneumonia [8].

In our work, we identified smoking status, job category, SARS-CoV-2 IgG serology status at enrollment, and the reported COVID-19 burden as risk factors for COVID-19. Risk factors reported in the literature for COVID-19 include sex [4,5,6,7,8], age [6,7,8,9], BMI [4,6,8], smoking status [4,8,42,43,44,45,46], and comorbidities, like higher glycated hemoglobin levels or use of blood pressure medications [4,8], asthma, or hypertension [4,5,8,10]. These risk factors were included in our analysis but not found to be statistically significant, which potentially could be related to the fact that only healthcare workers were included in our study. In our work, job category, SARS-CoV-2 IgG serology status at enrollment, and the reported COVID-19 burden were identified as the risk factors for both RTIs+COVID and COVID-19, indicating the same groups of participants at risk of contracting RTIs+COVID-19 or COVID-19.

Our study had several limitations. Firstly, only symptomatic events of COVID-19 cases diagnosed with a positive PCR or antigen test were used in the modeling, which potentially resulted in underreporting, because it was left to the participants to be tested or not. Additionally, while the model incorporated the reported COVID-19 burden, the hazard was not allowed to collapse to zero when the reported burden was approaching zero due to underreporting, especially at the beginning of the pandemic for which we have previously estimated the COVID-19 burden to be 2.76-fold higher than reported [29], likely because of the least amount of resources and diagnostics being available [47]. The extrapolation of the results in the future, outside of the context of the COVID-19 pandemic, must be done with care, as 25.7% of RTIs+COVID-19 events were confirmed as COVID-19, and this is likely a significant underestimation, as previously explored [27]. Lastly, data were collected during 2020 and 2021, including waves of the original strain, delta, and beta strains; thus, the results might be less representative of other COVID-19 waves.

In conclusion, nurses and doctors with obesity had the highest probability of developing RTIs+COVID-19. Non-smoking nurses and doctors had the highest probability of developing COVID-19, specifically. The reported COVID-19 burden increased event probability, while positive SARS-CoV-2 IgG serology at enrollment decreased the probability of RTIs+COVID-19 and COVID-19 specifically.

## Figures and Tables

**Table 1:** Participant demographics and covariate information included in the analysis. The same number of participants were present in both respiratory tract infections including COVID-19 (RTIs+COVID-19) and COVID-19 analyses.

Participant Characteristics	Unit	All Patients
Number of participants	n	1000
Gender (biological) female	n (%)	704 (70.4%)
Age (median [IQR])	years	39 (30–49)
BMI (median [IQR])	kg/m^2^	28.6 (24.1–34.6)
Job description	n (%)	
Nurse		165 (16.5%)
Doctor		144 (14.4%)
Essential worker		691 (69.1%)
Hypertension	n (%)	174 (17.4%)
Asthma	n (%)	68 (6.8%)
Diabetes mellitus	n (%)	63 (6.3%)
Cardiovascular disease	n (%)	24 (2.4%)
Self-reported smoker	n (%)	274 (27.4%)
Latent tuberculosis infection	n (%)	485 (48.5%)
Conversion from negative baseline to positive IGRA ^a^	n (%)	49 (4.9%)
Conversion from positive baseline to negative IGRA ^a^	n (%)	62 (6.2%)
Positive SARS-CoV-2 IgG serology at enrollment	n (%)	153 (15.3%)
Self-reported expected exposure to COVID-19 patients	n (%)	628 (62.8%)
South Africa District	n (%)	
Cape Town		950 (95.0%)
Garden Route		50 (5.0%)

N = number of participants, IQR = interquartile range, BMI = body mass index, IGRA = interferon-gamma release assay, SARS-CoV-2 = severe acute respiratory syndrome coronavirus 2, COVID-19 = coronavirus disease, BCG = Bacillus Calmette–Guérin. ^a^ Only the confirmed positive/negative IGRA status was considered when determining the participants who converted from negative at baseline to positive IGRA or converted from positive at baseline to negative IGRA. The remaining participants (who either did not convert or had missing or indeterminant IGRA status at either of the occasions) were considered as non-converters in the modeling.

**Table 2:** Final parameter estimates of the time-to-event model for respiratory tract infections including COVID-19 (RTIs+COVID-19) and COVID-19 (intention-to-treat analysis).

Parameter	Description	Estimate	RSE%	95% CI ^a^
Respiratory tract infections including COVID-19				
λ	Scale factor in the Gompertz function	2.679 × 10^−3^	10	2.282 × 10^−3^–3.189 × 10^−3^
α	Shape factor in the Gompertz function	−3.609 × 10^−3^	23	−4.467 × 10^−3^–−2.761 × 10^−3^
$\beta_{BURDEN}$	Reported COVID-19 burden influence on the hazard	0.237	13	0.178–0.296
$\beta_{JOB}$	Nurse/doctor job category influence on the hazard	0.434	20	0.262–0.599
$\beta_{SERO}$	Positive SARS-CoV-2 IgG serology at enrollment influence on the hazard	−0.539	26	−0.801–−0.269
$\beta_{BMI}$	BMI > 30 influence on the hazard	3.972 × 10^−2^	20	2.303 × 10^−2^–5.366 × 10^−2^
COVID-19				
λ	Scale factor in the Gompertz function	4.231 × 10^−4^	17	2.854 × 10^−4^–4.651 × 10^−4^
α	Shape factor in the Gompertz function	−3.666 × 10^−3^	22	−5.186 × 10^−3^–2.493 × 10^−3^
$\beta_{BURDEN}$	Reported COVID-19 burden influence on the hazard	0.669	6	0.577–0.753
$\beta_{JOB}$	Nurse/doctor job category influence on the hazard	0.515	29	0.226–0.814
$\beta_{SERO}$	Positive SARS-CoV-2 IgG serology at enrollment influence on the hazard	−2.580	23	−3.657–−1.553
$\beta_{SMOKING}$	Smoking influence on the hazard	−0.843	24	−1.257–−0.434

^a^ In the study, 95% confidence intervals (CI) were derived from sampling importance resampling with 2000 samples. λ = scale factor in the Gompertz function, α = shape factor in the Gompertz function, β = coefficient describing the risk factor, BURDEN = reported COVID-19 burden (per 1 case/10,000 capita increase), JOB = nurse/doctor job category, SERO = positive SARS-CoV-2 IgG serology at enrollment, SMOKING = self-reported smoker, BMI = body mass index (kg/m^2^) (per 1 kg/m^2^ increase), RSE = relative standard error, and CI = confidence interval.

## Data Availability

Data will be made available in an open data repository upon completion of all the secondary analyses. The study protocol and statistical analysis plan are available in the Upton et al. Supplementary Materials [27].

## Supplementary Materials

The following supporting information can be downloaded at: https://www.mdpi.com/article/10.3390/vaccines12030329/s1, Figure S1: Kaplan–Meier plots of data (enhanced *y*-axis) for the time-to-first event in the per-protocol analysis for (a) respiratory tract infections including COVID-19 (RTIs+COVID-19) and (b) COVID-19; Figure S2: visual predictive checks for the final models (enhanced *y*-axis) for the time-to-first event in the per-protocol analysis for (a) respiratory tract infections including COVID-19 (RTIs+COVID-19) and (b) COVID-19; Figure S3: forest plots for the statistically significant risk factor effects on cumulative probability in the per-protocol analysis for (a) respiratory tract infections including COVID-19 (RTIs+COVID-19) and (b) COVID-19; Figure S4: the cumulative probability of having an event within one year for different combinations of statistically significant risk factors in the per-protocol analysis for (a) respiratory tract infections including COVID-19 (RTIs+COVID-19) and (b) COVID-19; Figure S5: reported COVID-19 burden influence on the cumulative probability of having an event within one year for different combinations of statistically significant risk factors in the per-protocol analysis for (a) respiratory tract infections including COVID-19 (RTIs+COVID-19) and (b) COVID-19; Table S1: final parameter estimates of the time-to-event model for respiratory tract infections including COVID-19 (RTIs+COVID-19) and COVID-19 (per-protocol analysis); Text S1: final time-to-first RTIs including COVID-19 (RTIs+COVID-19) event model; Text S2: final time-to-first COVID-19 event model.
