# Supplementary material for: Risk Factors for COVID-19 and Respiratory Tract Infections during the Coronavirus Pandemic
Source: Vaccines (Basel). 2024 Mar 19;12(3):329. doi: 10.3390/vaccines12030329 (PMC10974083; doi:10.3390/vaccines12030329)
Supplement: Supplementary file 1 [file vaccines-12-00329-s001.zip › vaccines-2862857-supplementary.pdf]

Risk Factors for COVID-19 and Respiratory Tract Infections during the Coronavirus Pandemic

Laurynas Mockeliunas[a], Rob C. van Wijk[a], Caryn M. Upton[b], Jonathan Peter[c], Andreas H. Diacon[b], Ulrika S. H. Simonsson[a*]

Author affiliations:

[a]Department of Pharmaceutical Biosciences, Uppsala University, 751 24 Uppsala, Sweden

[b]TASK, Cape Town, South Africa

[c]Allergy and Immunology unit, University of Cape Town Lung Institute and Division of Allergy and Clinical Immunology, University of Cape Town, Cape Town, South Africa

***Correspondence**

Ulrika S. H. Simonsson

Department of Pharmaceutical Biosciences, Uppsala University, Box 591, 751 24 Uppsala, Sweden

Email: Ulrika.Simonsson@uu.se

Per-protocol analysis results

Respiratory tract infections including COVID-19

In the per-protocol analysis, 505 participants out of 1000 (50.5%) had an event. Kaplan-Meier plot of the data is shown in Figure S1, panel a. The baseline hazard function was best described by the Gompertz function, and the hazard decreased over time. A visual predictive check of the final model is shown in Figure S2, panel a. The same risk factors were identified compared to the intention-to-treat analysis. The final parameter estimates are presented in Table S1. The forest plot for statistically significant risk factors is shown in Figure S3, panel a, the cumulative probabilities within one year for different combinations of risk factors are shown in Figure S4, panel a, and the reported COVID-19 burden influence on the cumulative probability of an event within one year for different combinations of statistically significant risk factors is shown in Figure S5, panel a. The final model code is presented in Text S1.

COVID-19

In the per-protocol analysis, 169 out of 1000 participants (16.9%) reported COVID-19. Kaplan-Meier plot of the data is shown in Figure S1, panel b. The baseline hazard function was best described by the Gompertz function, and the hazard decreased over time. A visual predictive check of the final model is shown in Figure S2, panel b. The same risk factors were identified compared to the intention-to-treat analysis. The final parameter estimates are presented in Table S1. The forest plot for statistically significant risk factors is shown in Figure S3, panel b, the cumulative probabilities within one year for different combinations of risk factors are shown in Figure S4, panel b, and the reported COVID-19 burden in influence on the cumulative probability of an event within one year for different combinations of statistically significant risk factors is shown in Figure S5, panel b. The final model code is presented in Text S2.

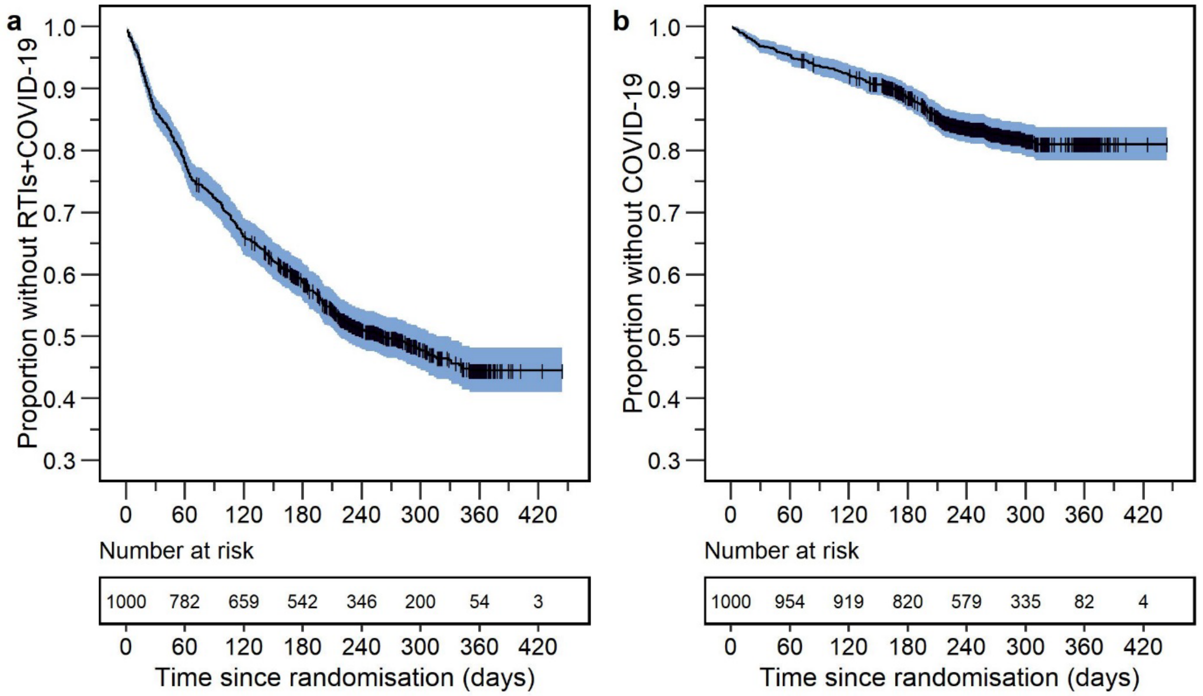

Figure S1. Kaplan–Meier plots of the data (enhanced y-axis) for the time-to-first event in the per-protocol analysis for (**a**) respiratory tract infections including COVID-19 (RTIs+COVID-19) and (**b**) COVID-19. The shaded area around the Kaplan–Meier curve represents the standard error, obtained by the Greenwood method [39]. Vertical dashes represent censoring, while a step down represents an event. The number at risk below the figure presents the number of participants still in the trial.

Table S1. Final parameter estimates of the time-to-event model for respiratory tract infections including COVID-19 (RTIs+COVID-19) and COVID-19 (per-protocol analysis).

Parameter	Description	Estimate	RSE%	95% CI[a]
	Respiratory tract infections including COVID-19			
λ	Scale factor in the Gompertz function	$2.897{\times}10^{-3}$	10	$2.424{\times}10^{-3}$–$3.418{\times}10^{-3}$
α	Shape factor in the Gompertz function	$-4.485{\times}10^{-3}$	13	$-5.318{\times}10^{-3}$–$-3.662{\times}10^{-3}$
β_{BURDEN}	Reported COVID-19 burden influence on the hazard	0.237	14	0.169–0.303
β_{JOB}	Nurse/Doctor job category influence on the hazard	0.404	25	0.238–0.581
β_{SERO}	Positive SARS-CoV-2 IgG serology at enrolment influence on the hazard	−0.549	38	−0.826–−0.268
β_{BMI}	BMI > 30 influence on the hazard	$3.932{\times}10^{-2}$	19	$2.238{\times}10^{-2}$–$5.689{\times}10^{-2}$
	COVID-19			
λ	Scale factor in the Gompertz function	$4.319{\times}10^{-4}$	18	$2.967{\times}10^{-4}$–$6.248{\times}10^{-4}$

α	Shape factor in the Gompertz function	-3.774×10^{-3}	28	-5.792×10^{-3}–-1.804×10^{-3}
β_{BURDEN}	Reported COVID-19 burden influence on the hazard	0.659	7	0.568–0.746
β_{JOB}	Nurse/Doctor job category influence on the hazard	0.563	28	0.255–0.865
β_{SERO}	Positive SARS-CoV-2 IgG serology at enrolment influence on the hazard	−2.805	26	−4.003–−1.703
$\beta_{SMOKING}$	Smoking influence on the hazard	−0.875	25	−1.325–−0.429

[a] In the study, 95% confidence intervals (CI) were derived from sampling importance resampling with 2000 samples. λ = scale factor in the Gompertz function, α = shape factor in the Gompertz function, β = coefficient describing the risk factor, BURDEN = reported COVID-19 burden (per 1 case/10,000 capita increase), JOB = nurse/doctor job category, SERO = positive SARS-CoV-2 IgG serology at enrollment, SMOKING = self-reported smoker, BMI = body mass index (kg/m^2) (per 1 kg/m^2 increase), RSE = relative standard error, and CI = confidence interval.

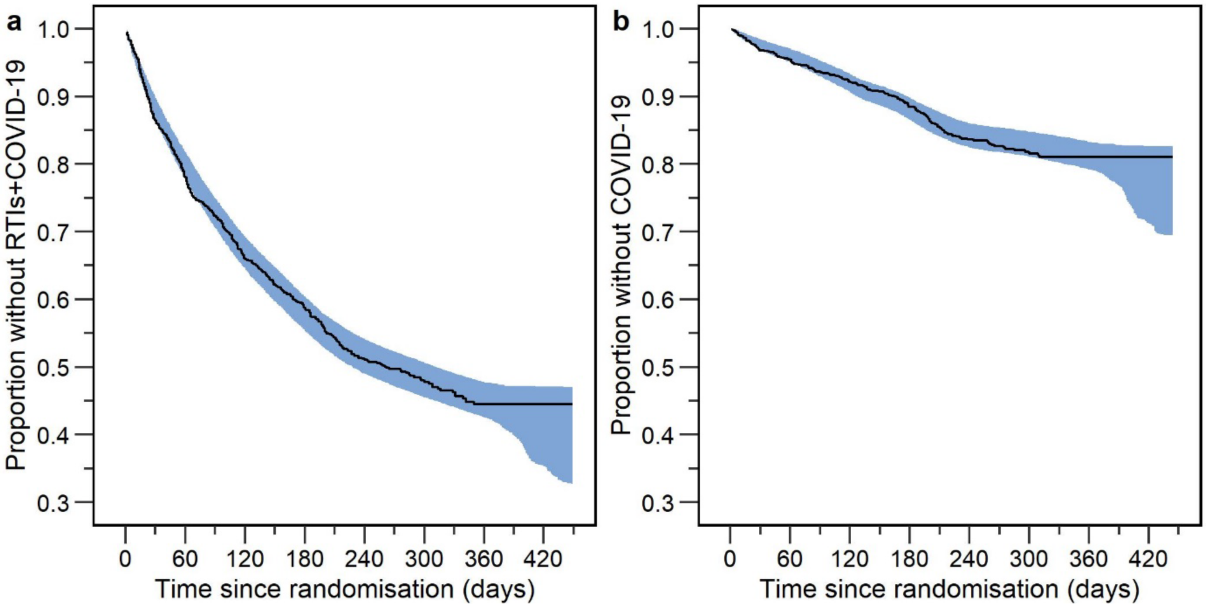

Figure S2. Visual predictive checks for the final models (enhanced y-axis) for the time-to-first event in the per-protocol analysis for (**a**) respiratory tract infections including COVID-19 (RTIs+COVID-19) and (**b**) COVID-19. The black line in the VPC represents observed data, while the shaded area represents the 95% confidence interval based on 1000 simulations using the final parametric time-to-event model.

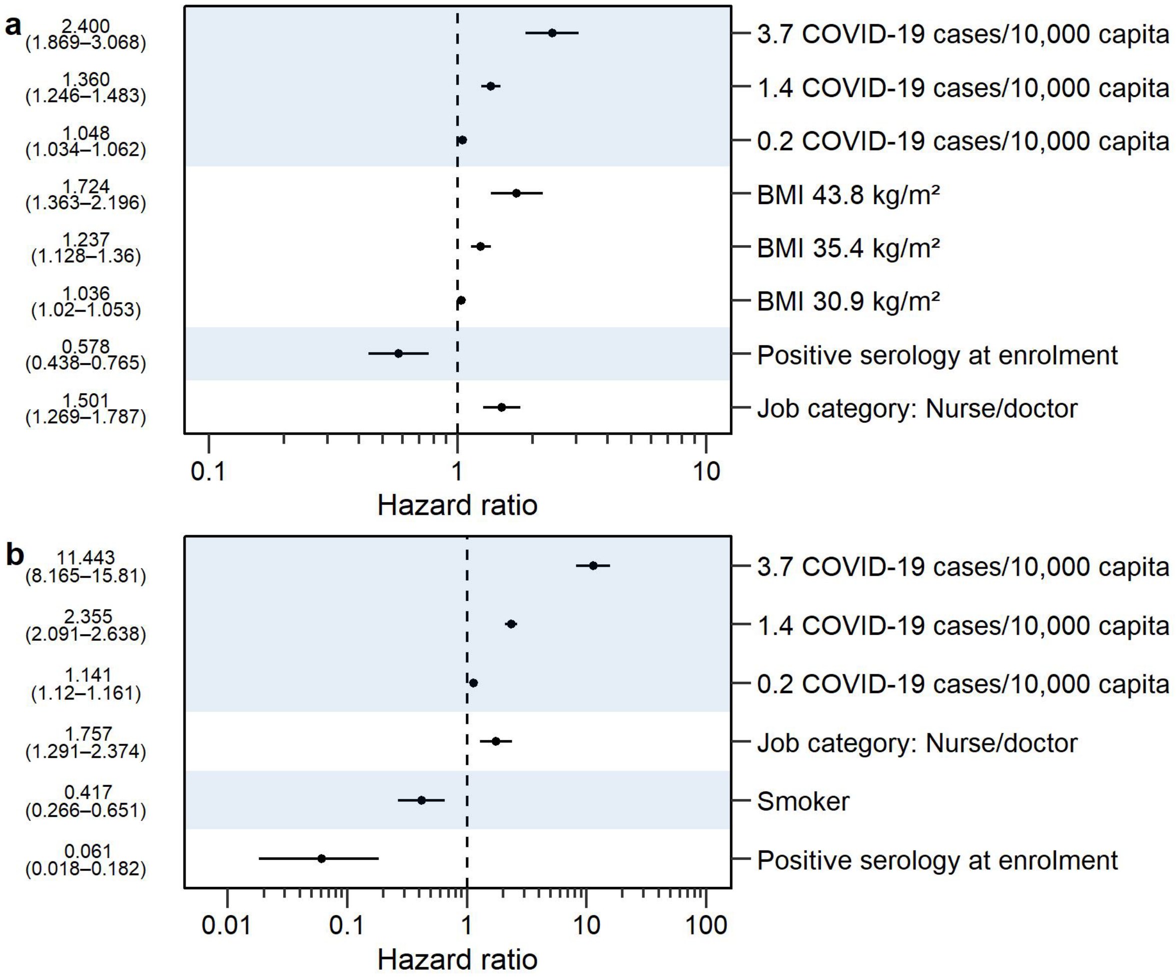

Figure S3. Forest plots for the statistically significant risk factor effects on cumulative probability in the per-protocol analysis for (**a**) respiratory tract infections including COVID-19 (RTIs+COVID-19) and (**b**) COVID-19. Risk factor effects are expressed as hazard ratios (HRs), the circle represents the median HR, while the whiskers represent a 95% confidence interval. The covariate values on the y-axis were derived from the observed data as either the unique categories of the categorical covariates or as the 10th, 50th, and 90th percentiles of the continuous covariates [40]. The numbers next to the forest plot represent the median HR and 95% confidence interval. Body mass index (BMI) < 30 kg/m^2 did not affect the hazard ratio while BMI > 30 kg/m^2 resulted in an increasing hazard ratio with an increasing covariate value.

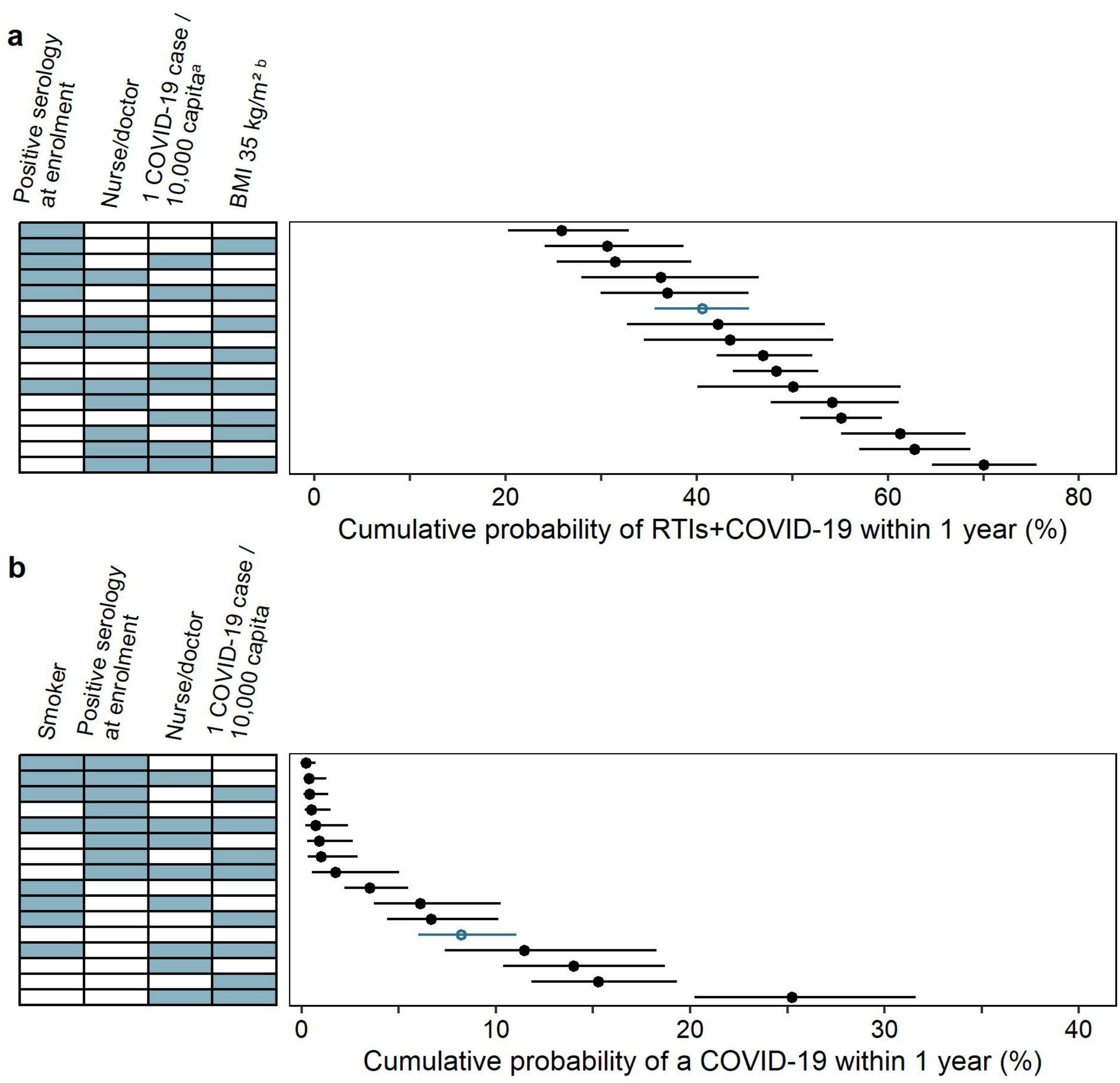

Figure S4. The cumulative probability of having an event within one year for different combinations of statistically significant risk factors in the per-protocol analysis for (**a**) respiratory tract infections including COVID-19 (RTIs+COVID-19) and (**b**) COVID-19. Blue cells represent the inclusion of a significant risk factor (named above), while white cells represent the inclusion of a reference feature (absence of the named risk factor). The circle represents the median, while the whiskers represent a 95% confidence interval. The reference cumulative probability (absence of any named risk factors) is shown in a blue empty circle. One COVID-19 case per 10,000 capita—reported COVID-19 burden, where the risk factor included was set to one case per 10,000 capita (reference: zero reported COVID-19 cases per 10,000 capita). Positive serology—corresponds to positive SARS-CoV-2 IgG serology at enrollment (reference: negative SARS-CoV-2 IgG serology at enrollment). Nurse/doctor—job category classified as a nurse/doctor (reference: essential worker). BMI 35 kg/m^2—body mass index (BMI) corresponding to being obese (reference: BMI < 30 kg/m^2). Smoker—indicates that the participant was a smoker (reference: non-smoker). [a] The continuous time-varying risk factor of the reported COVID-19 burden was treated as a categorical time-constant risk factor for the computation of cumulative probabilities, and two categories were selected: zero COVID-19 cases per 10,000 capita and one COVID-19 case per 10,000 capita. [b] The continuous covariate BMI was categorized into two categories for the purpose of computation

of cumulative probability: BMI < 30 kg/m^2 (indicating no impact of the risk factor on the cumulative probability) and BMI of 35 kg/m^2 (visualizing the impact of the risk factor on the cumulative probability).

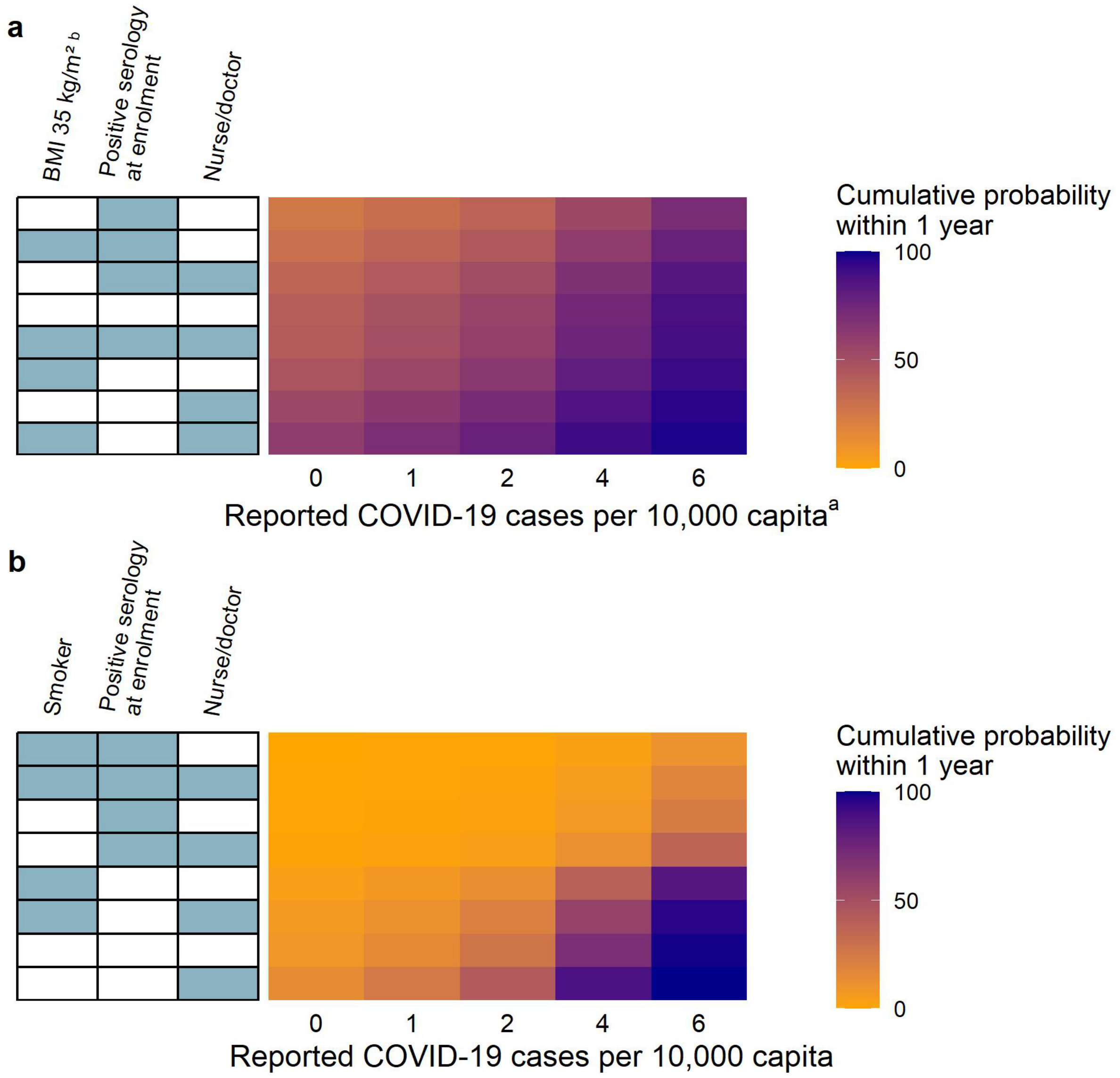

Figure S5. Reported COVID-19 burden influence on the cumulative probability of having an event within one year for different combinations of statistically significant risk factors in the per-protocol analysis for (**a**) respiratory tract infections including COVID-19 (RTIs+COVID-19) and (**b**) COVID-19. The heatmap shows the median cumulative probability for each combination of the reported COVID-19 burden and other statistically significant risk factors. The blue cells represent the inclusion of a significant risk factor (named above), while the white cells represent the inclusion of a reference feature (absence of the named risk factor). The circle represents the median, while the whiskers represent a 95% confidence interval. The reference cumulative probability (absence of any named risk factors) is shown in a blue empty circle. One COVID-19 case per 10,000 capita—reported COVID-19 burden, where the risk factor included was set to one case per 10,000 capita (reference: zero reported COVID-19 cases per 10,000 capita). Positive serology—corresponds to positive SARS-CoV-2 IgG serology at enrollment (reference: negative SARS-CoV-2 IgG serology at enrollment). Nurse/doctor—job category classified as a nurse/doctor (reference: essential worker). BMI of 35 kg/m^2—body mass index (BMI) corresponding to being obese (reference: BMI < 30 kg/m^2). Smoker—indicates that the participant was a smoker (reference: non-smoker).[a] The continuous time-varying risk factor of the reported COVID-19 burden was treated as a categorical time-constant risk factor for the computation of cumulative probabilities. [b] The

continuous covariate BMI was categorized into two categories for the purpose of computation of cumulative probability: BMI < 30 kg/m^2 and BMI of 35 kg/m^2.

Text S1. Final time-to-first RTIs including COVID-19 (RTI+COVID-19) event model

```
$PROBLEM Time-to-first RTI
$INPUT ID DV TIME EVID TYPE FTIME BMI JOB SERBASE BURDEN
; TYPE - internal filtering variable, 1 - time zero, 2 - records from time zero to event/censoring, 3 - event/censoring time, 4 - records for simulations
; JOB - job description, "Essential workers"=0, "Nurse"=1, "Doctor"=2
; BMI - Body mass index, unit=kg/m2
; SERBASE - serology status at enrollment, "Negative IgG serology at enrollment"=0, "Positive IgG serology at enrollment"=1
; FTIME - max follow-up time
; BURDEN - COVID-19 burden in South Africa (per 10,000 capita)

$DATA RTI_dataset.csv IGNORE=@
;Sim_start : remove from simulation model
 IGNORE=(TYPE.EQ.4)
;Sim_end
$SUBROUTINE ADVAN=6 TOL=9
$MODEL COMP=(HAZARD)
$PK
;;; RFSERBASE-DEFINITION START
IF(SERBASE.EQ.0) RFSERBASE = 0
IF(SERBASE.EQ.1) RFSERBASE = ( 0 + THETA(6))
;;; RFSERBASE-DEFINITION END

;;; RFBURDEN-DEFINITION START
RFBURDEN = ( 0 + THETA(5)*(BURDEN))
;;; RFBURDEN-DEFINITION END

;;; RFJOB-DEFINITION START
IF(JOB.EQ.0) RFJOB = 0
IF(JOB.NE.0) RFJOB = ( 0 + THETA(4))
;;; RFJOB-DEFINITION END
```

```
;;; RFBMI-DEFINITION START
RFBMI = 0
IF(BMI.GE.30) RFBMI = ( 0 + THETA(3)*(BMI - 30))
;;; RFBMI-DEFINITION END

;;; RF-RELATION START
RFCOV=RFBMI+RFJOB+RFBURDEN+RFSERBASE
;;; RF-RELATION END

LAM = THETA(1)*EXP(ETA(1))/100
SHP = THETA(2)

TVRF = 0

TVRF = RFCOV+TVRF
RF = 1*TVRF

$DES
DADT(1) = LAM*EXP(SHP*T)*EXP(RF)

$ERROR
IF(NEWIND.NE.2) OLDCHZ = 0
CHZ = A(1)-OLDCHZ
OLDCHZ = A(1)
SUR = EXP(-CHZ)
HAZNOW=LAM*EXP(SHP*TIME)*EXP(RF)
IF(DV.EQ.0) Y = SUR
IF(DV.NE.0) Y = SUR*HAZNOW

IF(ICALL.EQ.4) THEN
CALL RANDOM (2,R)
```

```
DV=0
RTTE = 0
IF(TIME.EQ.FTIME) RTTE = 1
IF(R.GT.SUR) THEN
DV=1
RTTE = 1
ENDIF
ENDIF

; ITT analysis final estimates
$THETA (0,0.2679) ; LAMBDA
$THETA -0.00361 ; ALPHA
$THETA 0.03972 ; RFBMI
$THETA 0.4341 ; RFJOB
$THETA 0.2379 ; RFBURDEN
$THETA -0.5385 ; RFSERBASE
$OMEGA 0 FIX

; PP analysis final estimates
;$THETA (0,0.2897) ; LAMBDA
;$THETA -0.004485 ; ALPHA
;$THETA 0.03932 ; RFBMI
;$THETA 0.4044 ; RFJOB
;$THETA 0.2367 ; RFBURDEN
;$THETA -0.5497 ; RFSERBASE
;$OMEGA 0 FIX

;Sim_start
$ESTIMATION MAXEVAL=9990 METHOD=0 LIKE PRINT=1 SIGL=9 NSIG=3
$COVARIANCE PRINT=E
;$SIMULATION (5988566) (39978 UNIFORM) ONLYSIM NOPREDICTION NSUB =
1000
```

Text S2. Final time-to-first COVID-19 event model

```
$PROBLEM Time-to-first COVID-19
$INPUT ID DV TIME EVID TYPE FTIME JOB SMOKING SERBASE BURDEN
; TYPE - internal filtering variable, 1 - time zero, 2 - records from time zero to event/censoring, 3 - event/censoring time, 4 - records for simulations
; JOB - job description, "Essential workers"=0, "Nurse"=1, "Doctor"=2
; SMOKING - smoking status, "Smoker"=1, "Non-smoker"=0
; SERBASE - serology status at enrollment, "Negative IgG serology at enrollment"=0, "Positive IgG serology at enrollment"=1
; FTIME - max follow-up time
; BURDEN - COVID-19 burden in South Africa (per 10,000 capita)

$DATA COVID19_dataset.csv IGNORE=@
;Sim_start : remove from simulation model
IGNORE=(TYPE.EQ.4)
;Sim_end

$SUBROUTINE ADVAN=6 TOL=9
$MODEL COMP=(HAZARD)
$PK
;;; RFSMOKING-DEFINITION START
IF(SMOKING.EQ.0) RFSMOKING = 0
IF(SMOKING.EQ.1) RFSMOKING = ( 0 + THETA(6))
;;; RFSMOKING-DEFINITION END

;;; RFSERBASE-DEFINITION START
IF(SERBASE.EQ.0) RFSERBASE = 0
IF(SERBASE.EQ.1) RFSERBASE = ( 0 + THETA(5))
;;; RFSERBASE-DEFINITION END

;;; RFBURDEN-DEFINITION START
RFBURDEN = ( 0 + THETA(4)*(BURDEN))
```

```
;;; RFBURDEN-DEFINITION END

;;; RFJOB-DEFINITION START
IF(JOB.EQ.0) RFJOB = 0
IF(JOB.NE.0) RFJOB = ( 0 + THETA(3))
;;; RFJOB-DEFINITION END

;;; RF-RELATION START
RFCOV=RFJOB+RFBURDEN+RFSERBASE+RFSMOKING
;;; RF-RELATION END

LAM = THETA(1)*EXP(ETA(1))/100
SHP = THETA(2)

TVRF = 0

TVRF = RFCOV+TVRF
RF = 1*TVRF

$DES
DADT(1) = LAM*EXP(SHP*T)*EXP(RF)

$ERROR
IF(NEWIND.NE.2) OLDCHZ = 0
CHZ = A(1)-OLDCHZ
OLDCHZ = A(1)
SUR = EXP(-CHZ)
HAZNOW=LAM*EXP(SHP*TIME)*EXP(RF)
IF(DV.EQ.0) Y = SUR
IF(DV.NE.0) Y = SUR*HAZNOW

 IF(ICALL.EQ.4) THEN
```

```
CALL RANDOM (2,R)
DV=0
RTTE = 0
IF(TIME.EQ.FTIME) RTTE = 1
IF(R.GT.SUR) THEN
DV=1
RTTE = 1
ENDIF
ENDIF

; ITT analysis final estimates
$THETA (0,0.04231) ; LAMBDA
$THETA -0.003666 ; ALPHA
$THETA 0.5151 ; RFJOB
$THETA 0.6688 ; RFBURDEN
$THETA -2.58 ; RFSERBASE
$THETA -0.8432 ; RFSMOKING
$OMEGA 0 FIX

; PP analysis final estimates
;$THETA (0,0.04319) ; LAMBDA
;$THETA -0.003774 ; ALPHA
;$THETA 0.5629 ; RFJOB
;$THETA 0.6591 ; RFBURDEN
;$THETA -2.805 ; RFSERBASE
;$THETA -0.8752 ; RFSMOKING
;$OMEGA 0 FIX

;Sim_start
$ESTIMATION MAXEVAL=9990 METHOD=0 LIKE PRINT=1 SIGL=9 NSIG=3
$COVARIANCE PRINT=E
```

;$SIMULATION (5988566) (39978 UNIFORM) ONLYSIM NOPREDICTION NSUB = 1000

;Sim_end
